# Pulmonary Histoplasmosis, Taiwan, 1997–2024 (Response)

**DOI:** 10.3201/eid3209.261249

**Published:** 2026-09

**Authors:** Ting-Wei Kao, Shang-Chen Yang, Hsiang-Wei Hu, Yu-Tsung Huang, Chin-Chung Shu, Wang-Huei Sheng

**Affiliations:** Author affiliations: National Taiwan University Hospital, Department of Internal Medicine, National Taiwan University College of Medicine, Taipei, Taiwan (T.-W. Kao, S.-C. Yang, C-.C. Shu, W.-H. Sheng); National Taiwan University Hospital, Department of Pathology, Taipei (H.-W. Hu); National Taiwan University Hospital, Department of Laboratory Medicine, National Taiwan University College of Medicine, Taipei (Y.-T. Huang); Graduate Institute of Clinical Medicine, National Taiwan University College of Medicine, Taipei (C.-C. Shu); National Taiwan University Hospital Hsinchu Branch, Department of Internal Medicine, Hsinchu, Taiwan (W.-H. Sheng)

**Keywords:** histoplasmosis, dimorphic fungi, fungi, *Histoplasma capsulatum*, non-endemic regions, pulmonary mycosis, Taiwan

**In Response:** We sincerely thank Lin et al*.* for their insightful comments on our article (*1*) and for providing valuable molecular epidemiologic evidence regarding *Histoplasma capsulatum* in Taiwan. The identification of a Latin American A1 lineage isolate in a patient without a travel history raises interesting questions regarding the local epidemiology of histoplasmosis. Their report highlights the increasing role of molecular approaches in understanding transmission dynamics and phylogeography of *Histoplasma* spp. fungi. Such molecular characterization might represent a necessary direction for future epidemiologic investigations. However, our retrospective study spanned nearly 3 decades, during which fungal isolates were not routinely preserved for molecular characterization. Consequently, comparable genomic data were unavailable for most patients.

The case report shared notable similarities with our findings. Both studies identified patients with histoplasmosis without a travel history, supporting the possibility of local acquisition or previously unrecognized environmental exposure. Also, both studies demonstrate that histoplasmosis is not restricted to immunocompromised persons and should not be regarded only as an imported infection in Taiwan. The report used molecular analysis to characterize a fungal isolate obtained from culture. In contrast, fungal cultures were infrequently positive in our cohort, highlighting one of the major diagnostic challenges encountered in routine clinical practice.

Whether these cases reflect local environmental reservoirs, historical introduction events, or previously unrecognized transmission pathways remains unclear. Expanded environmental sampling, preservation of fungal isolates, and molecular surveillance are needed to clarify the geographic distribution, lineage diversity, and transmission ecology of *Histoplasma* spp. fungi in Taiwan and other traditionally nonendemic regions. That limitation also could contribute to underrecognition of locally acquired histoplasmosis.
